# Supplementary material for: Toolbox Accelerating Glycomics (TAG): Improving Large-Scale Serum Glycomics and Refinement to Identify SALSA-Modified and Rare Glycans
Source: Int J Mol Sci. 2022 Oct 28;23(21):13097. doi: 10.3390/ijms232113097 (PMC9656093; doi:10.3390/ijms232113097)
Supplement: Supplementary file 1 [file ijms-23-13097-s001.zip › Figure S1 Biosynthetic pathway for trimming of high-mannose glycans and the synthesis of hybrid glycans.pptx]

## Slide 1
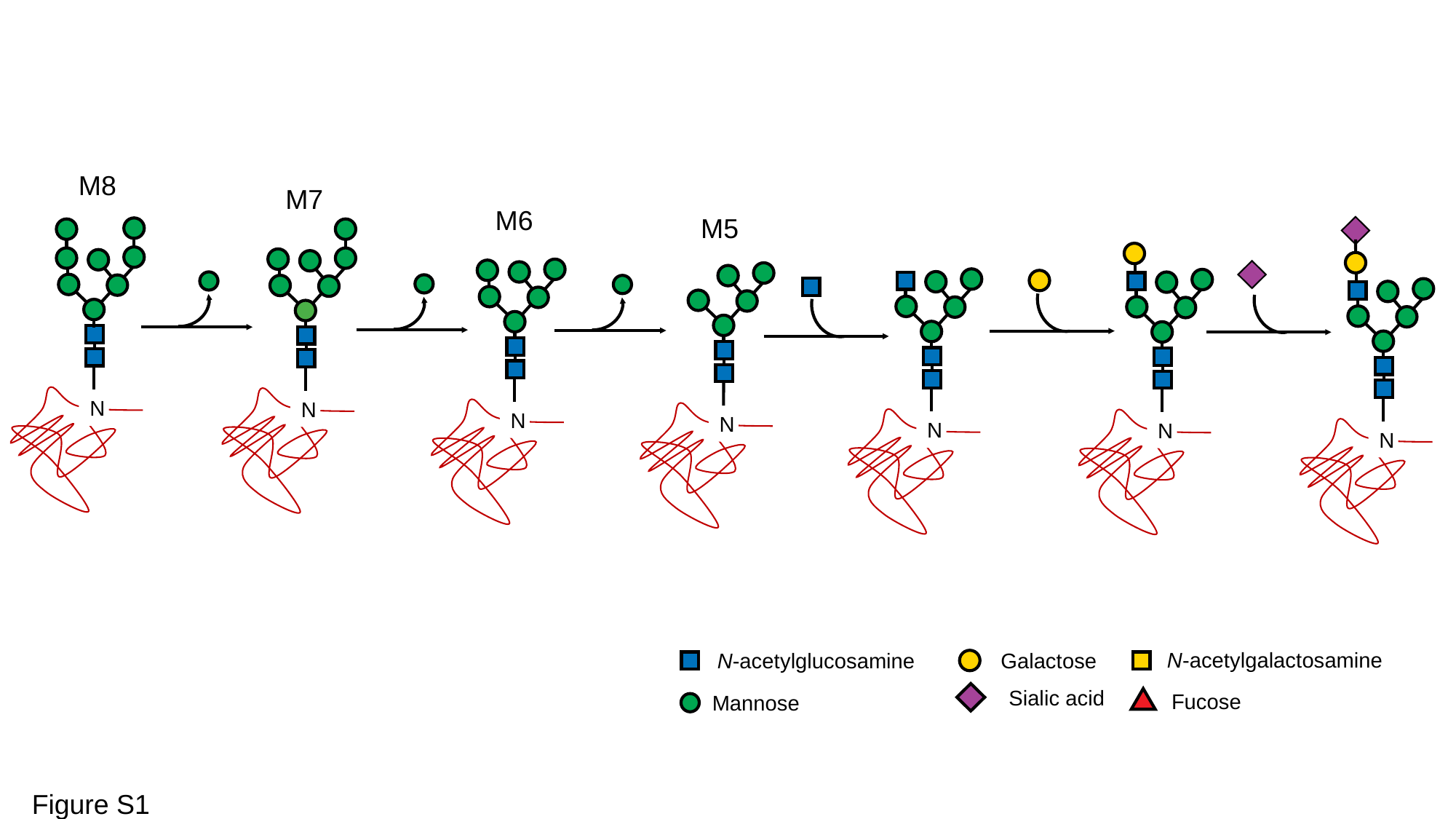

M8
M7
M6
M5
N
N
N
N
N
N
N
N-acetylgalactosamine
N-acetylglucosamine
Galactose
Sialic acid
Mannose
Fucose
Figure S1
