# Supplementary material for: Toolbox Accelerating Glycomics (TAG): Improving Large-Scale Serum Glycomics and Refinement to Identify SALSA-Modified and Rare Glycans
Source: Int J Mol Sci. 2022 Oct 28;23(21):13097. doi: 10.3390/ijms232113097 (PMC9656093; doi:10.3390/ijms232113097)

Glycan quantity for each experiment (calibration curve)

1(6): *m/z*= 1118.3750
(Hex)6
600000000000000

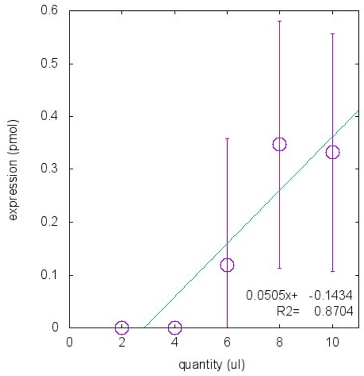

2(18): *m/z*= 1362.4808
(Hex)2 + (Man)3(GlcNAc)2
200000000000000C

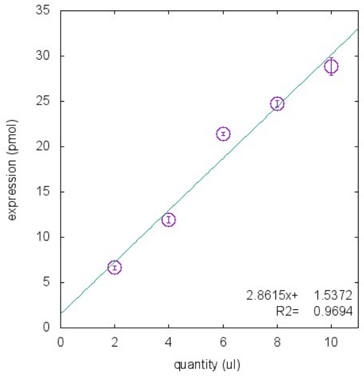

3(19): *m/z*= 1524.5336
(Hex)3 + (Man)3(GlcNAc)2
300000000000000C

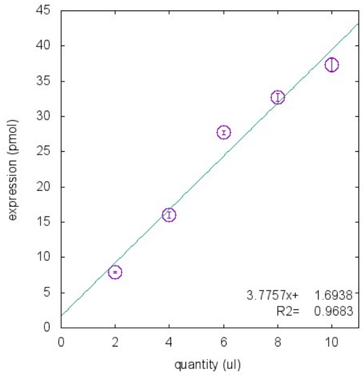

4(20): *m/z*= 1686.5864
(Hex)4 + (Man)3(GlcNAc)2
400000000000000C

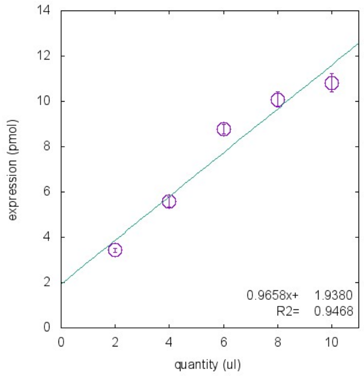

5(21): *m/z*= 1848.6392
(Hex)5 + (Man)3(GlcNAc)2
500000000000000C

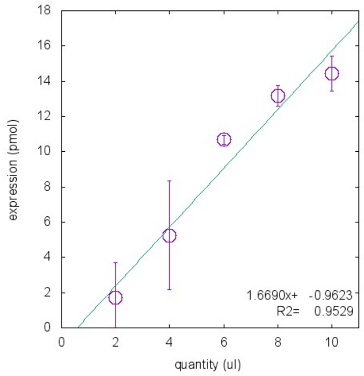

6(22): *m/z*= 2010.6920
(Hex)6 + (Man)3(GlcNAc)2
600000000000000C

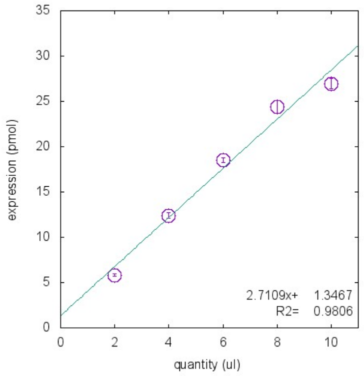

7(27): *m/z*= 1444.5338
(HexNAc)2 +
(Man)3(GlcNAc)2
020000000000000C

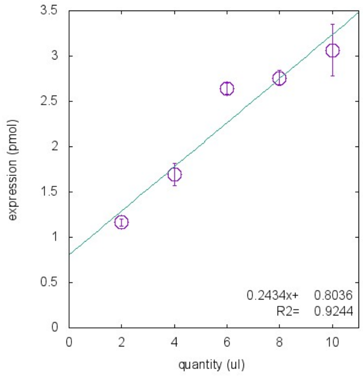

8(28): *m/z*= 1647.6131
(HexNAc)3 +
(Man)3(GlcNAc)2
030000000000000C

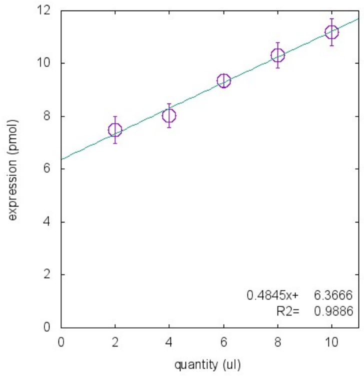

9(31): *m/z*= 1403.5073
(Hex)1 (HexNAc)1 +
(Man)3(GlcNAc)2
110000000000000C

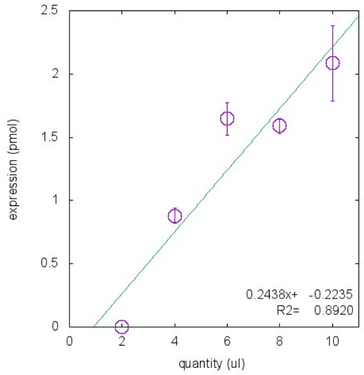

10(32): *m/z*= 1606.5866
(Hex)1 (HexNAc)2 +
(Man)3(GlcNAc)2
120000000000000C

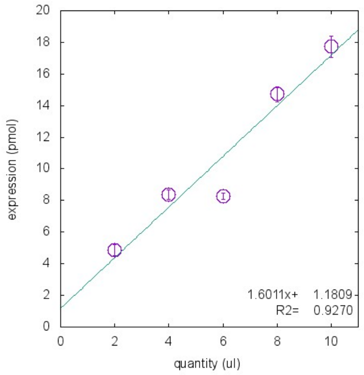

11(33): *m/z*= 1809.6659
(Hex)1 (HexNAc)3 +
(Man)3(GlcNAc)2
130000000000000C

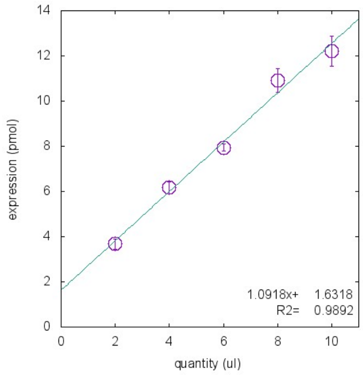

12(36): *m/z*= 1565.5601
(Hex)2 (HexNAc)1 +
(Man)3(GlcNAc)2
210000000000000C

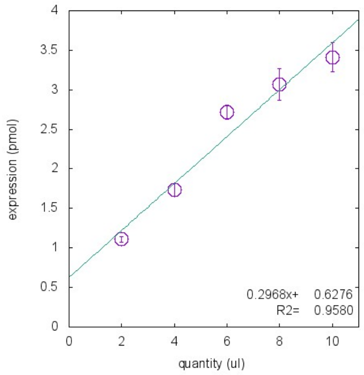

13(37): *m/z*= 1768.6394
(Hex)2 (HexNAc)2 +
(Man)3(GlcNAc)2
220000000000000C

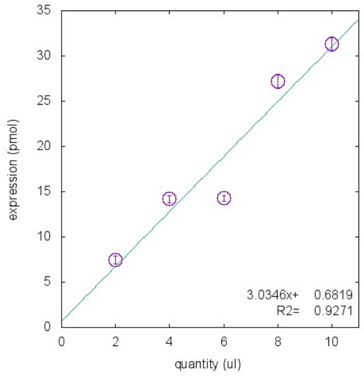

14(38): *m/z*= 1971.7187
(Hex)2 (HexNAc)3 +
(Man)3(GlcNAc)2
230000000000000C

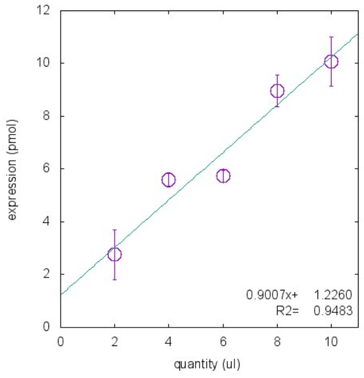

15(41): *m/z*= 1727.6129
(Hex)3 (HexNAc)1 +
(Man)3(GlcNAc)2
310000000000000C

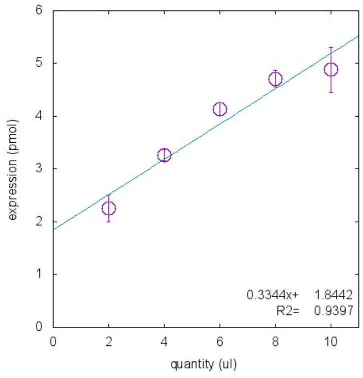

16(62): *m/z*= 1590.5917
(HexNAc)2 (Fuc)1 +
(Man)3(GlcNAc)2
021000000000000C

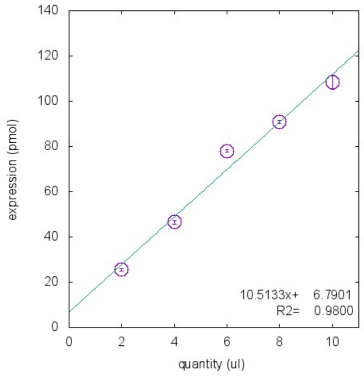

17(63): *m/z*= 1793.6710
(HexNAc)3 (Fuc)1 +
(Man)3(GlcNAc)2
031000000000000C

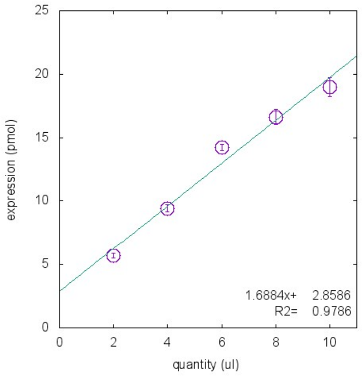

18(67): *m/z*= 1752.6445
(Hex)1 (HexNAc)2 (Fuc)1 +
(Man)3(GlcNAc)2
121000000000000C

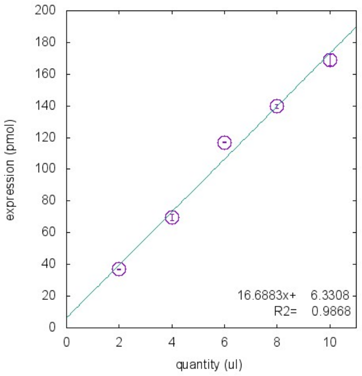

19(68): *m/z*= 1955.7238
(Hex)1 (HexNAc)3 (Fuc)1 +
(Man)3(GlcNAc)2
131000000000000C

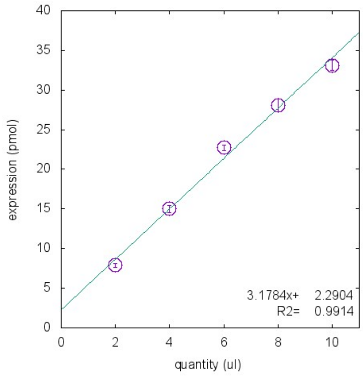

20(72): *m/z*= 1914.6973
(Hex)2 (HexNAc)2 (Fuc)1 +
(Man)3(GlcNAc)2
221000000000000C

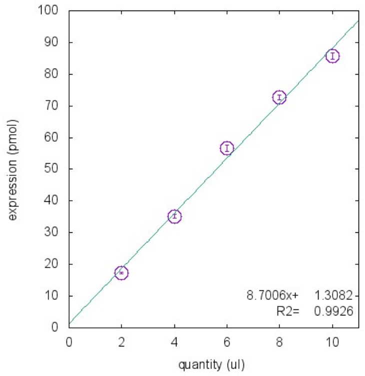

21(141): *m/z*= 2327.8394
(Hex)4 (HexNAc)1 (Fuc)3 +
(Man)3(GlcNAc)2
413000000000000C

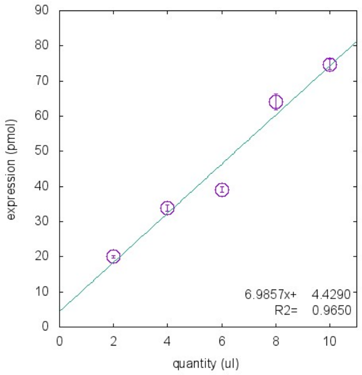

22(147): *m/z*= 2692.9715
(Hex)5 (HexNAc)2 (Fuc)3 +
(Man)3(GlcNAc)2
523000000000000C

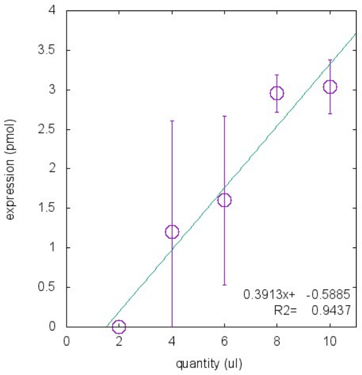

23(148): *m/z*= 2896.0508
(Hex)5 (HexNAc)3 (Fuc)3 +
(Man)3(GlcNAc)2
533000000000000C

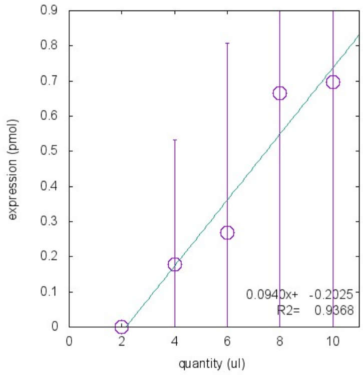

24(165): *m/z*= 2432.9195
(Hex)1 (HexNAc)5 (GlcA)1
+ (Man)3(GlcNAc)2
150000000000010C

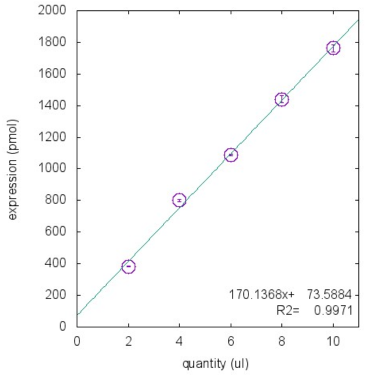

25(193): *m/z*= 2010.7660
(HexNAc)3 (Fuc)1 (GlcA)1
+ (Man)3(GlcNAc)2
031000000000010C

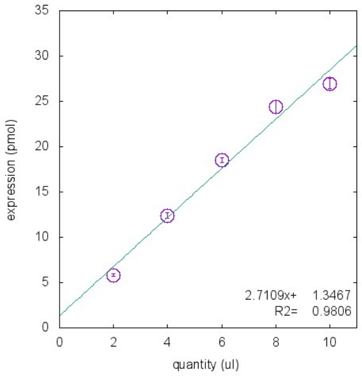

26(200): *m/z*= 2578.9774
(Hex)1 (HexNAc)5 (Fuc)1
(GlcA)1 +
(Man)3(GlcNAc)2
151000000000010C

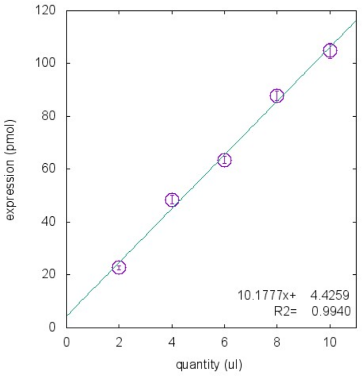

27(216): *m/z*= 2414.8714
(Hex)5 (HexNAc)1 (Fuc)1
(GlcA)1 +
(Man)3(GlcNAc)2
511000000000010C

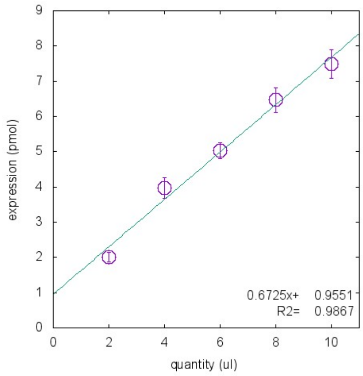

28(237): *m/z*= 2439.9030
(Hex)3 (HexNAc)2 (Fuc)2
(GlcA)1 +
(Man)3(GlcNAc)2
322000000000010C

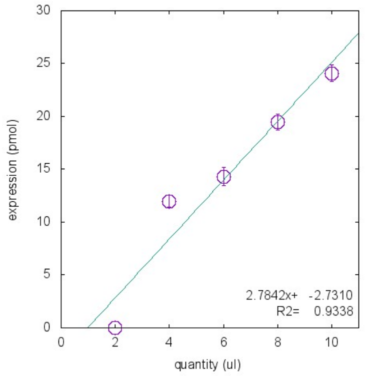

29(286): *m/z*= 1573.6128
(HexNAc)1 (6NeuAc)1 +
(Man)3(GlcNAc)2
010000100000000C

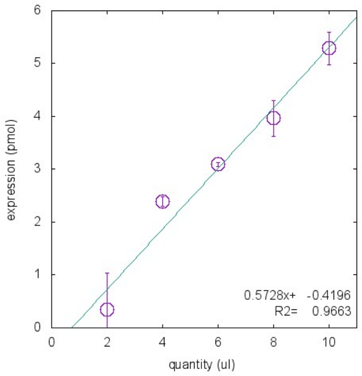

30(296): *m/z*= 1735.6656
(Hex)1 (HexNAc)1
(6NeuAc)1 +
(Man)3(GlcNAc)2
110000100000000C

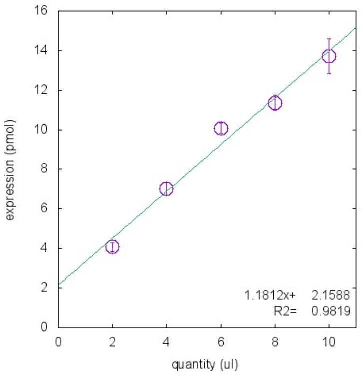

31(297): *m/z*= 1707.6343
(Hex)1 (HexNAc)1
(3-8NeuAc)1 +
(Man)3(GlcNAc)2
110001000000000C

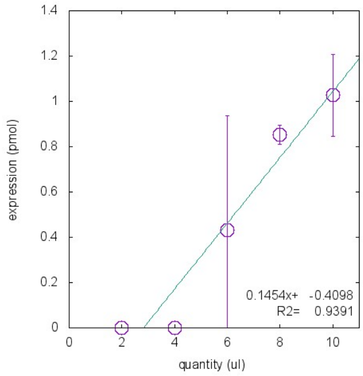

32(298): *m/z*= 1938.7449
(Hex)1 (HexNAc)2
(6NeuAc)1 +
(Man)3(GlcNAc)2
120000100000000C

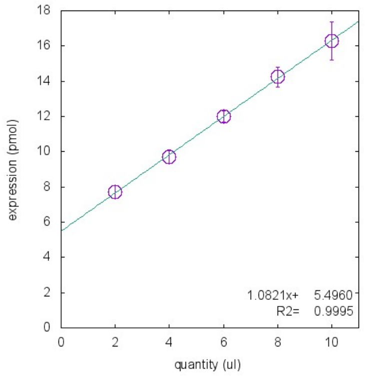

33(300): *m/z*= 2141.8242
(Hex)1 (HexNAc)3
(6NeuAc)1 +
(Man)3(GlcNAc)2
130000100000000C

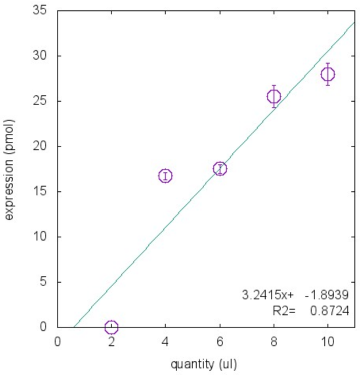

34(306): *m/z*= 1897.7184
(Hex)2 (HexNAc)1
(6NeuAc)1 +
(Man)3(GlcNAc)2
210000100000000C

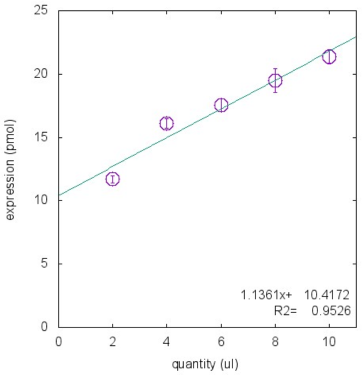

35(307): *m/z*= 1869.6871
(Hex)2 (HexNAc)1
(3-8NeuAc)1 +
(Man)3(GlcNAc)2
210001000000000C

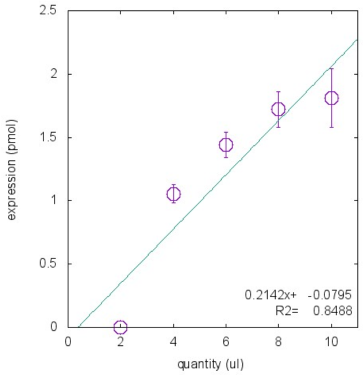

36(308): *m/z*= 2100.7977
(Hex)2 (HexNAc)2
(6NeuAc)1 +
(Man)3(GlcNAc)2
220000100000000C

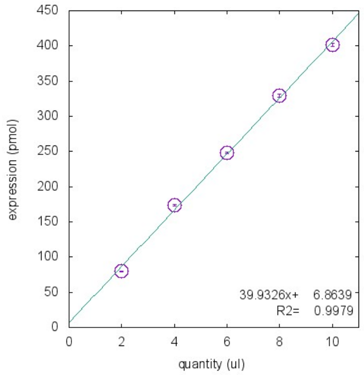

37(309): *m/z*= 2072.7664
(Hex)2 (HexNAc)2
(3-8NeuAc)1 +
(Man)3(GlcNAc)2
220001000000000C

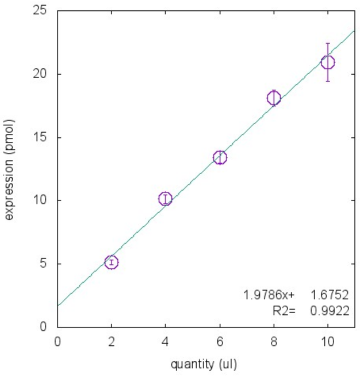

38(310): *m/z*= 2303.8770
(Hex)2 (HexNAc)3
(6NeuAc)1 +
(Man)3(GlcNAc)2
230000100000000C

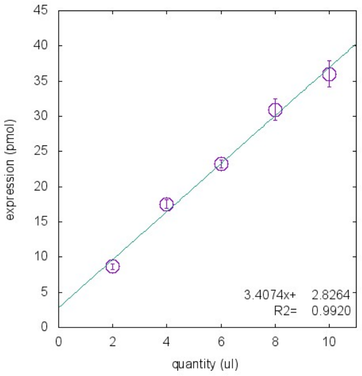

39(316): *m/z*= 2059.7712
(Hex)3 (HexNAc)1
(6NeuAc)1 +
(Man)3(GlcNAc)2
310000100000000C

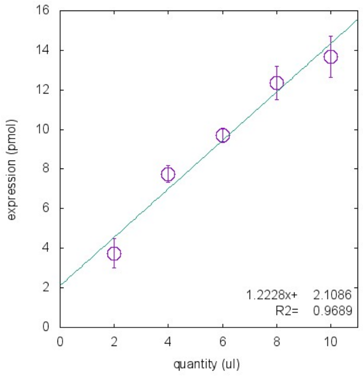

40(317): *m/z*= 2031.7399
(Hex)3 (HexNAc)1
(3-8NeuAc)1 +
(Man)3(GlcNAc)2
310001000000000C

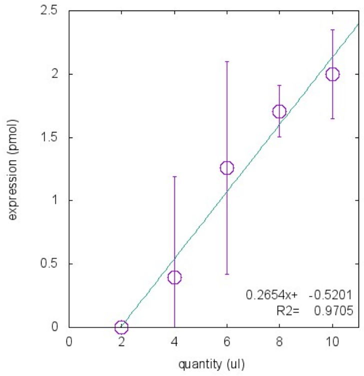

41(320): *m/z*= 2465.9298
(Hex)3 (HexNAc)3
(6NeuAc)1 +
(Man)3(GlcNAc)2
330000100000000C

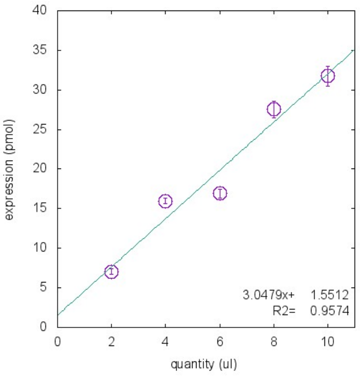

42(332): *m/z*= 2831.0619
(Hex)4 (HexNAc)4
(6NeuAc)1 +
(Man)3(GlcNAc)2
440000100000000C

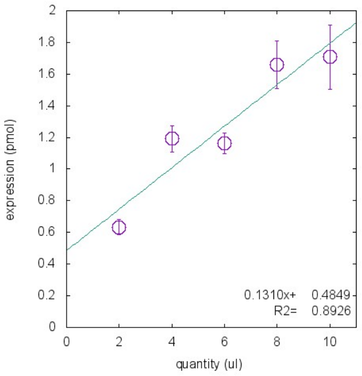

43(336): *m/z*= 2383.8768
(Hex)5 (HexNAc)1
(6NeuAc)1 +
(Man)3(GlcNAc)2
510000100000000C

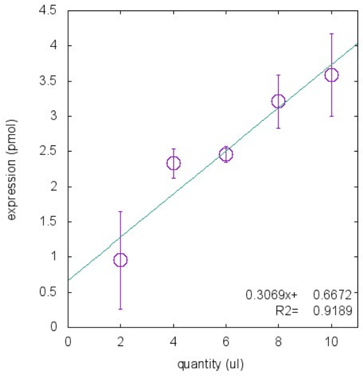

44(368): *m/z*= 2084.8028
(Hex)1 (HexNAc)2 (Fuc)1
(6NeuAc)1 +
(Man)3(GlcNAc)2
121000100000000C

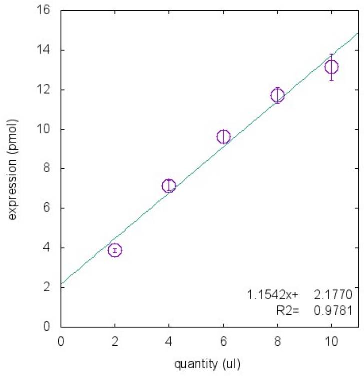

45(370): *m/z*= 2287.8821
(Hex)1 (HexNAc)3 (Fuc)1
(6NeuAc)1 +
(Man)3(GlcNAc)2
131000100000000C

46(372): *m/z*= 2490.9614
(Hex)1 (HexNAc)4 (Fuc)1
(6NeuAc)1 +
(Man)3(GlcNAc)2
141000100000000C

47(378): *m/z*= 2246.8556
(Hex)2 (HexNAc)2 (Fuc)1
(6NeuAc)1 +
(Man)3(GlcNAc)2
221000100000000C

48(379): *m/z*= 2218.8243
(Hex)2 (HexNAc)2 (Fuc)1
(3-8NeuAc)1 +
(Man)3(GlcNAc)2
221001000000000C

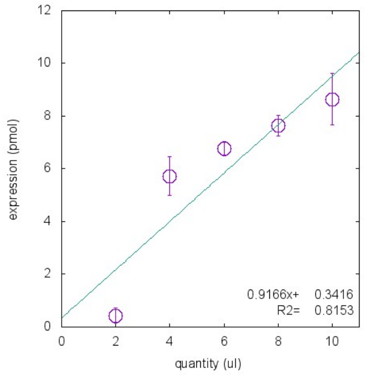

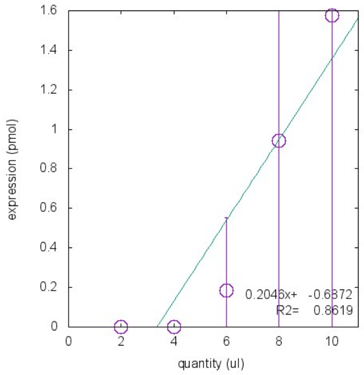

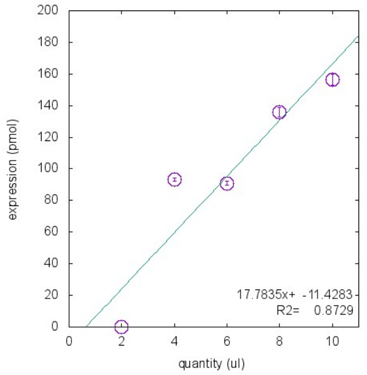

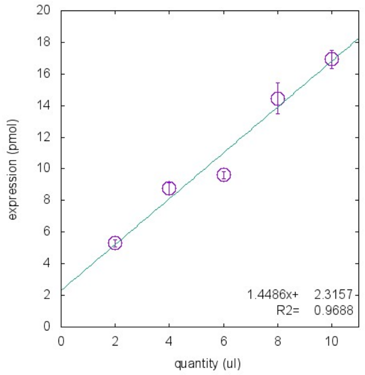

49(390): *m/z*= 2611.9877
(Hex)3 (HexNAc)3 (Fuc)1
(6NeuAc)1 +
(Man)3(GlcNAc)2
331000100000000C

50(397): *m/z*= 2339.8506
(Hex)4 (HexNAc)1 (Fuc)1
(3-8NeuAc)1 +
(Man)3(GlcNAc)2
411001000000000C

51(428): *m/z*= 2474.9665
(HexNAc)4 (Fuc)2
(6NeuAc)1 +
(Man)3(GlcNAc)2
042000100000000C

52(500): *m/z*= 2742.0507
(Hex)2 (HexNAc)3 (Fuc)3
(6NeuAc)1 +
(Man)3(GlcNAc)2
233000100000000C

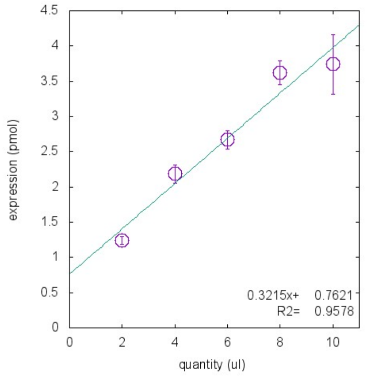

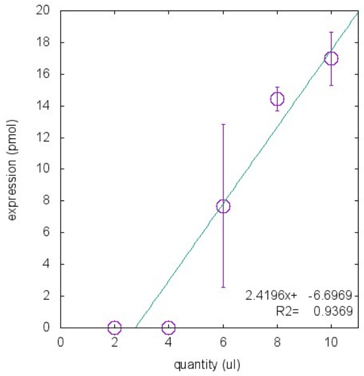

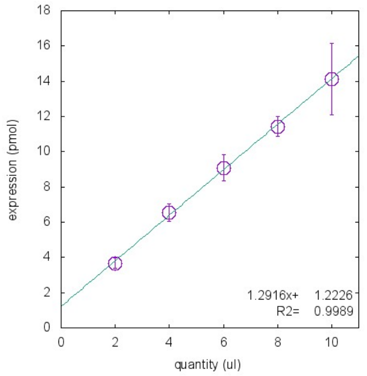

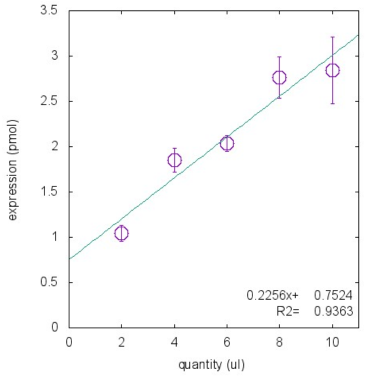

53(529): *m/z*= 2997.0985
(Hex)5 (HexNAc)2 (Fuc)3
(3-8NeuAc)1 +
(Man)3(GlcNAc)2
523001000000000C

54(554): *m/z*= 2317.8927
(Hex)2 (HexNAc)2
(6NeuAc)1 (GlcA)1 +
(Man)3(GlcNAc)2
220000100000010C

55(561): *m/z*= 2899.0993
(Hex)2 (HexNAc)5
(3-8NeuAc)1 (GlcA)1 +
(Man)3(GlcNAc)2
250001000000010C

56(609): *m/z*= 3045.1572
(Hex)2 (HexNAc)5 (Fuc)1
(3-8NeuAc)1 (GlcA)1 +
(Man)3(GlcNAc)2
251001000000010C

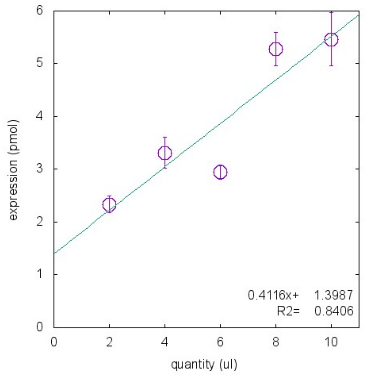

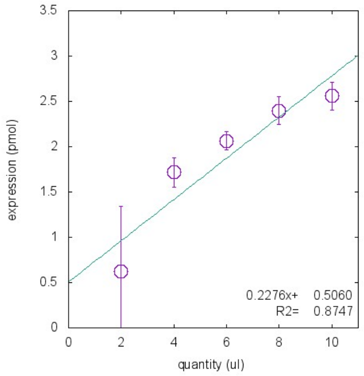

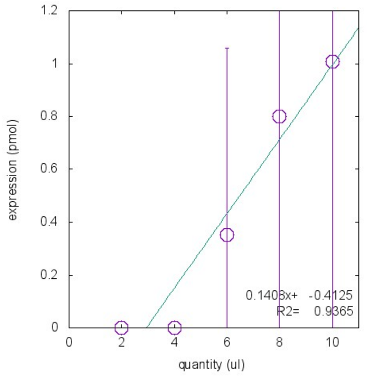

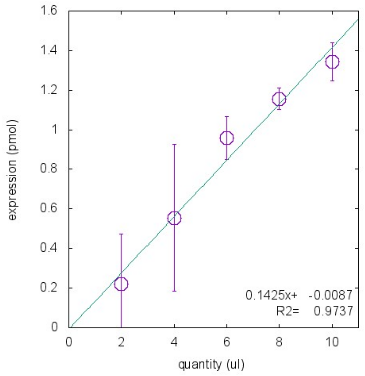

57(736): *m/z*= 2677.0618
(Hex)1 (HexNAc)4
(6NeuAc)2 +
(Man)3(GlcNAc)2
140000200000000C

58(742): *m/z*= 2432.9560
(Hex)2 (HexNAc)2
(6NeuAc)2 +
(Man)3(GlcNAc)2
220000200000000C

59(743): *m/z*= 2404.9247
(Hex)2 (HexNAc)2
(3-8NeuAc)1 (6NeuAc)1 +
(Man)3(GlcNAc)2
220001100000000C

60(744): *m/z*= 2376.8934
(Hex)2 (HexNAc)2
(3-8NeuAc)2 +
(Man)3(GlcNAc)2
220002000000000C

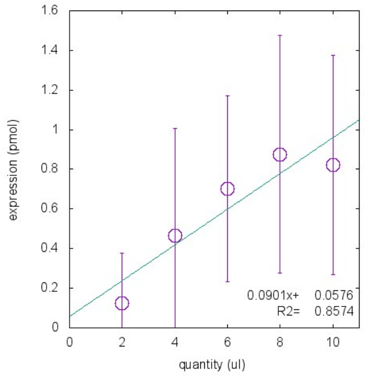

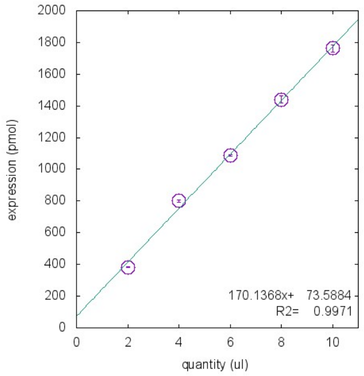

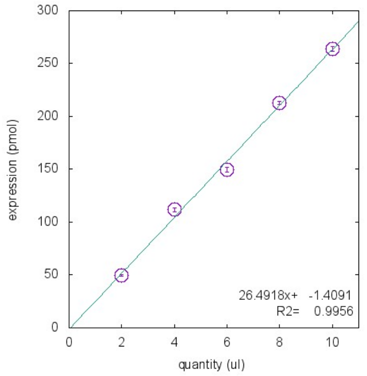

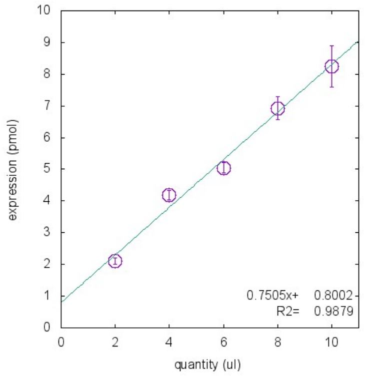

61(745): *m/z*= 2636.0353
(Hex)2 (HexNAc)3
(6NeuAc)2 +

62(755): *m/z*= 2566.9775
(Hex)3 (HexNAc)2
(3-8NeuAc)1 (6NeuAc)1 +

63(757): *m/z*= 2798.0881
(Hex)3 (HexNAc)3
(6NeuAc)2 +

64(758): *m/z*= 2770.0568
(Hex)3 (HexNAc)3
(3-8NeuAc)1 (6NeuAc)1 +

(Man)3(GlcNAc)2
230000200000000C

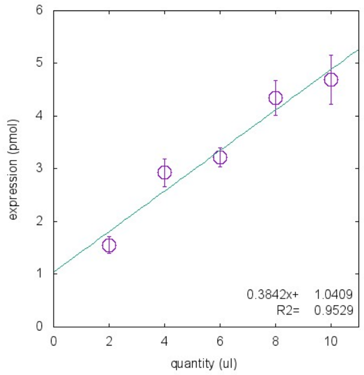

(Man)3(GlcNAc)2
320001100000000C

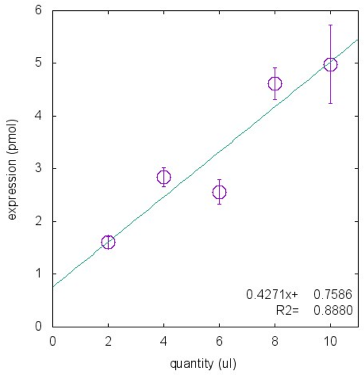

(Man)3(GlcNAc)2
330000200000000C

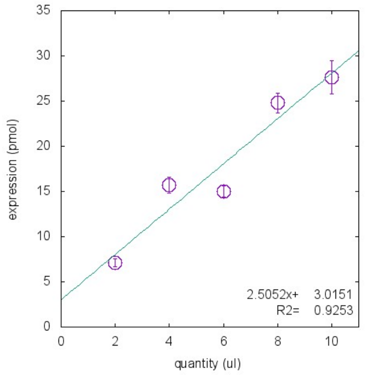

(Man)3(GlcNAc)2
330001100000000C

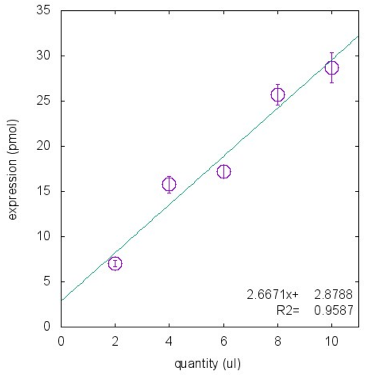

65(759): *m/z*= 2742.0255
(Hex)3 (HexNAc)3
(3-8NeuAc)2 +
(Man)3(GlcNAc)2
330002000000000C

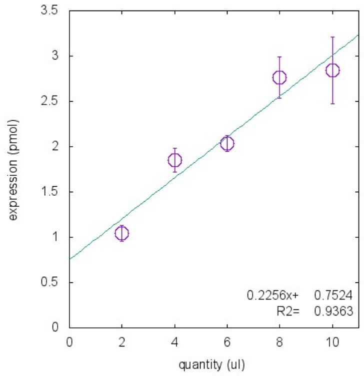

66(773): *m/z*= 3135.1889
(Hex)4 (HexNAc)4
(3-8NeuAc)1 (6NeuAc)1 +
(Man)3(GlcNAc)2
440001100000000C

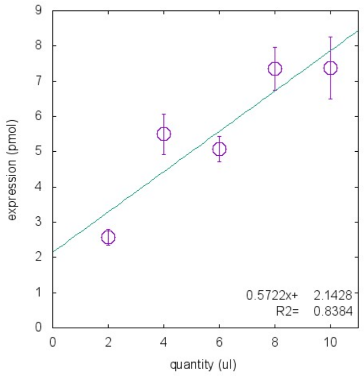

67(814): *m/z*= 2579.0139
(Hex)2 (HexNAc)2 (Fuc)1
(6NeuAc)2 +
(Man)3(GlcNAc)2
221000200000000C

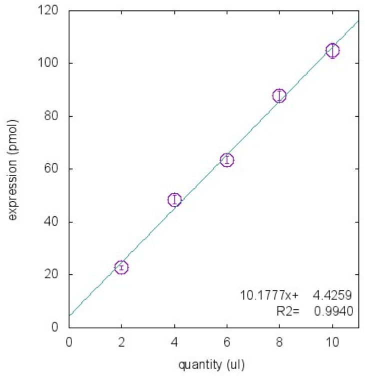

68(815): *m/z*= 2550.9826
(Hex)2 (HexNAc)2 (Fuc)1
(3-8NeuAc)1 (6NeuAc)1 +
(Man)3(GlcNAc)2
221001100000000C

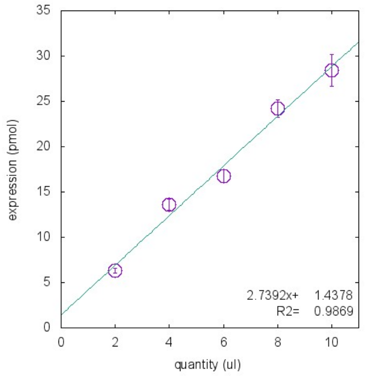

69(816): *m/z*= 2522.9513
(Hex)2 (HexNAc)2 (Fuc)1
(3-8NeuAc)2 +
(Man)3(GlcNAc)2
221002000000000C

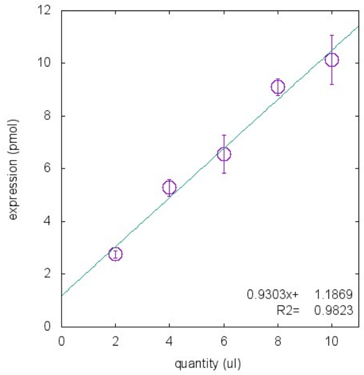

70(817): *m/z*= 2782.0932
(Hex)2 (HexNAc)3 (Fuc)1
(6NeuAc)2 +
(Man)3(GlcNAc)2
231000200000000C

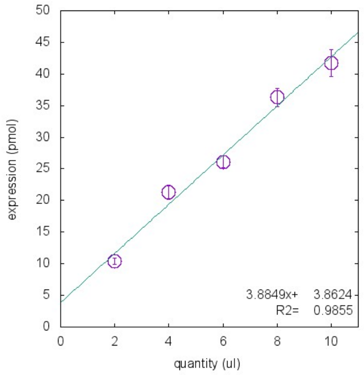

71(818): *m/z*= 2754.0619
(Hex)2 (HexNAc)3 (Fuc)1
(3-8NeuAc)1 (6NeuAc)1 +
(Man)3(GlcNAc)2
231001100000000C

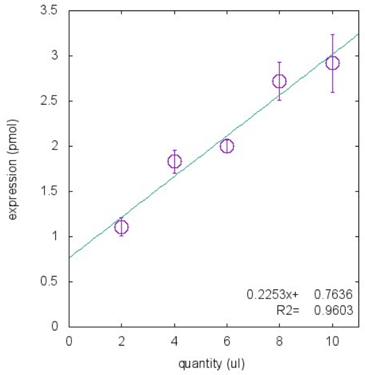

72(829): *m/z*= 2944.1460
(Hex)3 (HexNAc)3 (Fuc)1
(6NeuAc)2 +
(Man)3(GlcNAc)2
331000200000000C

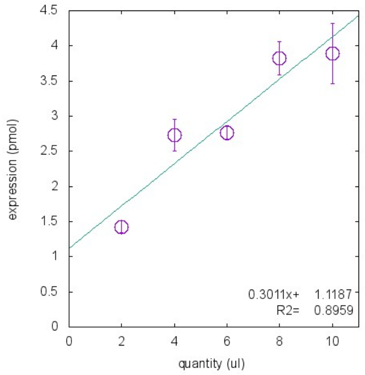

73(830): *m/z*= 2916.1147
(Hex)3 (HexNAc)3 (Fuc)1
(3-8NeuAc)1 (6NeuAc)1 +
(Man)3(GlcNAc)2
331001100000000C

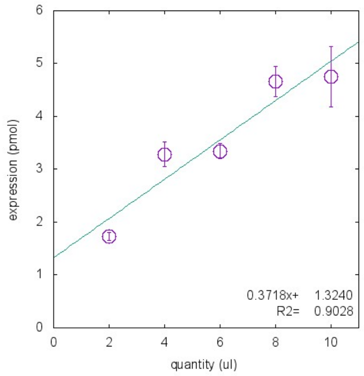

74(894): *m/z*= 2831.0620
(Hex)3 (HexNAc)2 (Fuc)2
(3-8NeuAc)2 +
(Man)3(GlcNAc)2
322002000000000C

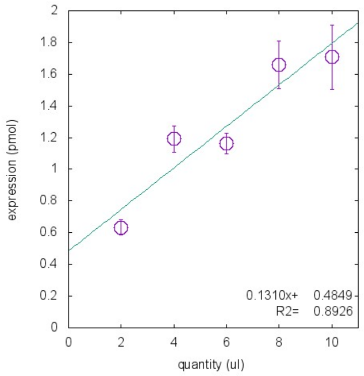

75(949): *m/z*= 3074.2090
(Hex)2 (HexNAc)3 (Fuc)3
(6NeuAc)2 +
(Man)3(GlcNAc)2
233000200000000C

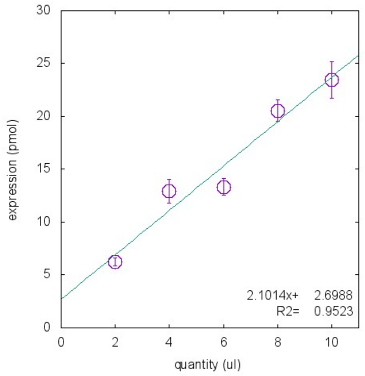

76(1198): *m/z*= 3130.2464
(Hex)3 (HexNAc)3
(6NeuAc)3 +
(Man)3(GlcNAc)2
330000300000000C

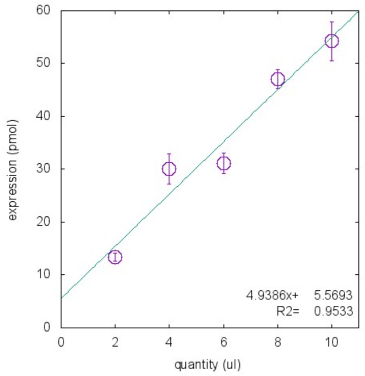

77(1199): *m/z*= 3102.2151
(Hex)3 (HexNAc)3
(3-8NeuAc)1 (6NeuAc)2 +
(Man)3(GlcNAc)2
330001200000000C

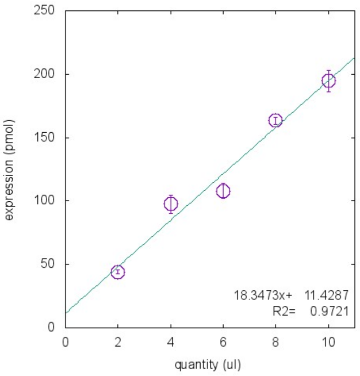

78(1200): *m/z*= 3074.1838
(Hex)3 (HexNAc)3
(3-8NeuAc)2 (6NeuAc)1 +
(Man)3(GlcNAc)2
330002100000000C

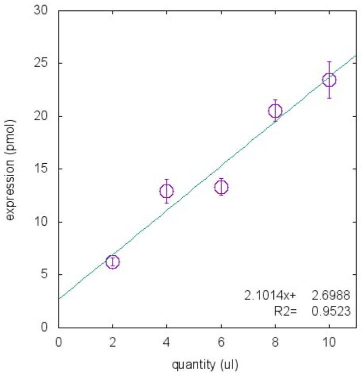

79(1259): *m/z*= 3248.2730
(Hex)3 (HexNAc)3 (Fuc)1
(3-8NeuAc)1 (6NeuAc)2 +
(Man)3(GlcNAc)2
331001200000000C

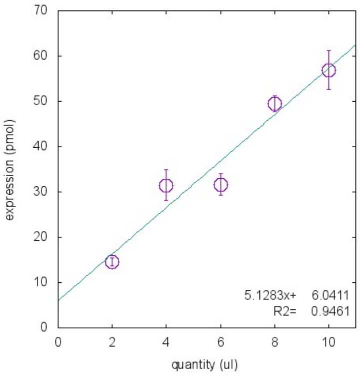

80(1260): *m/z*= 3220.2417
(Hex)3 (HexNAc)3 (Fuc)1
(3-8NeuAc)2 (6NeuAc)1 +
(Man)3(GlcNAc)2
331002100000000C

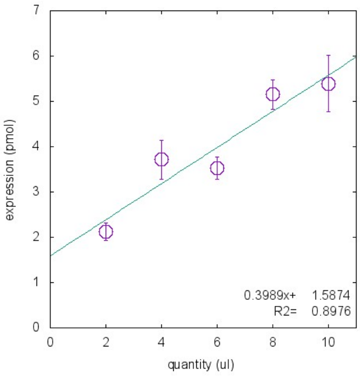

81(1261): *m/z*= 3192.2104
(Hex)3 (HexNAc)3 (Fuc)1
(3-8NeuAc)3 +
(Man)3(GlcNAc)2
331003000000000C

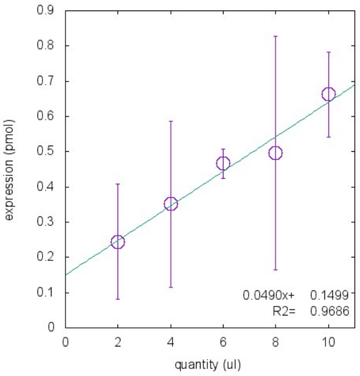

Supplement: Supplementary file 1 [file ijms-23-13097-s001.zip › Figure S2 Calibration curves in the serum analysis.pdf]
