# Supplementary material for: Toolbox Accelerating Glycomics (TAG): Improving Large-Scale Serum Glycomics and Refinement to Identify SALSA-Modified and Rare Glycans
Source: Int J Mol Sci. 2022 Oct 28;23(21):13097. doi: 10.3390/ijms232113097 (PMC9656093; doi:10.3390/ijms232113097)
Supplement: Supplementary file 1 [file ijms-23-13097-s001.zip › Table S1 Complete list of glycan residues in TAG.pdf]

Table S1. Complete list of glycan residues in TAG. In parentheses, we described the modification method of carboxylic acid of sialic acid.

	**Name**	**Description**
1	Hex	Hexose
2	HexNAc	*N*-acetyl hexose
3	Fuc	Fucose
4	NeuAc	*N*-acetylneuraminic acid (methylesterification)
5	NeuGc	*N*-glycolylneuraminic acid (methylesterification)
6	3-8NeuAc	α2,3- or α2,8-linkage of NeuAc (SALSA)
7	6NeuAc	α2,6-linkage of NeuAc (SALSA)
8	3-8NeuGc	α2,3- or α2,8-linkage of NeuGc (SALSA)
9	6NeuGc	α2,6-linkage of NeuGc (SALSA)
10	KDN	2-keto-3-deoxynonoic acid (methylesterification)
11	HSO3	Sulfation modification
12	Lactone	Lactonization modification of sialic acids
13	Ac	Acetylation modification
14	GlcA	Glucuronic acid (isopropylamine amination)
15	P	Phosphorylation modification
