# Supplementary material for: Toolbox Accelerating Glycomics (TAG): Improving Large-Scale Serum Glycomics and Refinement to Identify SALSA-Modified and Rare Glycans
Source: Int J Mol Sci. 2022 Oct 28;23(21):13097. doi: 10.3390/ijms232113097 (PMC9656093; doi:10.3390/ijms232113097)
Supplement: Supplementary file 1 [file ijms-23-13097-s001.zip › Table S2 All major output files from TAG Expression.pdf]

Table S2. All major output files from TAG Expression

File name	Description
Summary.csv	The summary file of the analysis containing the observed mass, the expression level estimated from the area, its mean value, standard deviation, CV, and the URL to search the attributed structure against Glyconnect for each identified glycan. See main text for details.
exp_list.csv	This file contains the same data as out_list.csv but slightly abbreviated. The data include expression levels of glycans, mean, standard deviation, CV value, t-value, *p*-value, and the total for each type. The total for each type is also shown in the lower part of the table, but it can only be used as a rough guide since some types are double-counted because all possible structures are attributed in TAG.
exp_list_zerocut.csv	In addition to exp_list.csv, glycans with zero abundance for all experiments are removed and displayed in a compact format. As for exp_list.csv, the total of each type is also shown at the bottom, but it can only be used as a rough guide since some types are double-counted because all possible structures are attributed in TAG.
each_glycan_quant.html	Expression levels of attributed glycans are summarized in bar graphs for each series. This is in html format, so it can be viewed with ordinary web browsers.
each_glycan_quant_point.html	Data are compiled into a scatter plot for calibration curves. Linear fitting is performed, and a, b, and R^2 are also output. Data are in html format and can be viewed with ordinary web browsers.
each_glycan_quant_point_rcut.html	Only glycans with R^2 >0.8 are shown in the scatter plot for the calibration curve. Linear fitting is performed, and a, b, and R^2 are also output. Data are in html format and can be viewed with ordinary web browsers.
